# Clonal Hematopoiesis and Liquid Biopsy in Gastrointestinal Cancers

**DOI:** 10.3389/fmed.2021.772166

**Published:** 2022-01-21

**Authors:** Vlad M. Croitoru, Irina M. Cazacu, Ionut Popescu, Doru Paul, Simona Olimpia Dima, Adina Emilia Croitoru, Alina Daniela Tanase

**Affiliations:** ^1^Faculty of Medicine, Titu Maiorescu University, Bucharest, Romania; ^2^Department of Medical Oncology, Fundeni Clinical Institute, Bucharest, Romania; ^3^Division of Hematology and Medical Oncology, Department of Medicine, Weill Cornell Medicine/New York-Presbyterian, New York, NY, United States; ^4^Center of Excellence in Translational Medicine, Fundeni Clinical Institute, Bucharest, Romania; ^5^Bone Marrow Transplant Unit, Fundeni Clinical Institute, Bucharest, Romania

**Keywords:** clonal hematopoiesis, liquid biopsy, gastrointestinal tumor, cancer, cfDNA

## Abstract

The use of blood liquid biopsy is increasingly being incorporated into the clinical setting of gastrointestinal cancers care. Clonal hematopoiesis (CH) occurs naturally as a result of the accumulation of somatic mutations and the clonal proliferation of hematopoietic stem cells with normal aging. The identification of CH-mutations has been described as a source of biological noise in blood liquid biopsy. Incorrect interpretation of CH events as cancer related can have a direct impact on cancer diagnosis and treatment. This review summarizes the current understanding of CH as a form of biological noise in blood liquid biopsy and the reported clinical significance of CH in patients with GI cancers.

## Introduction

Gastrointestinal (GI) cancers contribute significantly to cancer-related burden (1). Given the high mortality of GI cancers, the field of GI malignancies is in high need of a better understanding of tumor biology.

Tissue biopsy is currently the gold standard for cancer diagnosis and allows tumor classification and molecular profiling. It also provides insight into prognosis, and guides treatment strategy. However, repeated tissue biopsies are difficult to obtain, and intratumoral heterogeneity limits the ability to achieve timely and accurate tumor characterization (2). Liquid biopsies can overcome these issues and represents a less invasive and more extensive approach to cancer diagnosis, therapy choice and treatment response monitoring.

It has been clearly demonstrated that liquid biopsies can detect molecular changes associated with GI tumors in body fluids (3). Peripheral blood remains the most studied material for liquid biopsy as it contains viable circulating tumor cells (CTCs) and circulating tumor DNA (ctDNA) released from apoptotic or necrotic tumor cells. Cell free DNA (cfDNA) are nucleic acid fragments that enter the bloodstream during apoptosis or necrosis; however, a significant amount of cfDNA comes from normal cells (4). Thus, the analysis of cfDNA in cancer patients is limited by the low quantity of molecules released by the cancer cells into the bloodstream, and by the presence of circulating DNA from normal cells (5).

The presence of various somatic mutations in plasma remains a challenge for the precise interpretation of liquid biopsy results. CH represents a process in which somatic mutations occurring in hematopoietic stem cells leads to clonal expansion of these mutations in blood cells. Thus, CH may be responsible for the non-cancer mutations detected in cfDNA (6).

CH is part of the normal aging process and is common in the general population (7). The genes that are most commonly mutated in CH are *DNMT3A, TET2, ASXL1*, and *JAK2* (8). However, CH mutations can also affect genes related to cancer such as *KRAS, PIK3CA and EGFR* (9–11). These mutations from hematopoietic cells could be a source of biological noise in cfDNA analysis. Misclassification of CH mutations as tumor-related could lead to inappropriate decisions for patient management (6).

CH origins from hematopoietic stem cells and most often lead to differentiation bias toward the myeloid compartment. This is important as these myeloid cells are the source of CH signals in liquid biopsies. Despite the fact that mutations had various growth patterns, the highest proportion of expansion consistently occurred in the stem cell compartment indicative for early clonal dominance. The two most frequently mutated genes in CH, DNMT3A and TET2, show diverse patterns of hematopoietic cell involvement and a distinct myeloid proliferation bias (12, 13). TET2 mutations cause an increase in myeloid proliferation. The majority of DNMT3A mutations occur in multipotent stem cells, and lineage-specific proliferation is regulated by many intrinsic/extrinsic variables.

In the review, we have summarized the current understanding of CH as a form of biological noise in blood liquid biopsy and the reported clinical significance of CH in patients with GI cancers.

## The Emerging Role of Liquid Biopsy in Gastrointestinal Cancers

The use of liquid biopsy as a tool to enhance screening/early detection, disease monitoring, and disease recurrence in GI malignancies is a fast-developing area.

Liquid biopsy has been evaluated for **early cancer detection**. Certain blood tests that can improve screening and early detection have been authorized for clinical use (14) or are currently being developed (15). CancerSEEK (10) is a blood test that utilizes ctDNA and protein biomarkers to classify cancer into eight different types with various sensitivities and specificities. Although further prospective trials in bigger populations are required, preliminary findings suggest that this test may be useful for cancer screening.

Also, the use of ctDNA for surveillance of residual disease and recurrence has been investigated. Curative surgery remains a critical component of therapy for patients with early-stage GI malignancies. ctDNA has been shown to provide advantages over imaging in evaluating minimal residual disease (MRD) after curative surgery in colorectal cancer (16, 17). Enhanced identification of MRD in these patients may help better adjuvant therapy selection. Liquid biopsy has also been extensively investigated as a technique for monitoring disease recurrence, and serial profiling has enabled identification of recurrence prior to radiological imaging in patients with colorectal cancer and pancreatic cancer (3, 18). Numerous studies (19, 20) demonstrated a decrease in ctDNA levels in response to chemotherapy and a subsequent increase in case of treatment resistance.

Additionally, liquid biopsies have shown significant clinical usefulness as a prognostic tool for GI malignancies (3). Yao et al. (21) identified the presence of RAS/BRAF mutations in ctDNA as a predictive factor for poor progression-free survival in metastatic colorectal cancer patients receiving first-line treatment.

Another use of liquid biopsy is to discover therapeutic targets, predict therapy response, and monitor the development of tumor resistance over time. Garlan et al. showed that variations in ctDNA concentration may be used to predict the effectiveness of first- or second-line treatment in a prospective cohort of 82 metastatic colorectal cancer patients (22).

Treatment resistance patterns in colorectal cancer, especially to epidermal growth factor receptor (EGFR) inhibition, have been extensively investigated. ctDNA analysis demonstrated a high sensitivity for identifying mutations in *KRAS, BRAF, and EGFR* (23, 24). Siravegna et al. (23) studied clonal evolution using ctDNA throughout therapy with the EGFR inhibitors cetuximab and panitumumab. They observed the development of *KRAS*-mutated clones (and therefore disease progression) after anti-EGFR antibody therapy, followed by a subsequent decrease in these *KRAS*-mutated clonal populations after the removal of the EGFR blockade. Additionally, these findings support the usefulness of re-challenging with EGFR-inhibitors after salvage chemotherapy—a concept whose clinical effectiveness has been also validated. Clearly, long term monitoring of ctDNA for the purpose of modifying and adapting treatment methods has significant therapeutic value.

## Clonal Hematopoiesis: Biological Noise in Liquid Biopsy?

Non-invasive detection of tumor-associated mutations in the blood represents an important clinical tool. However, analysis of cfDNA is challenging, as mutations occur at very low frequencies and can be difficult to interpret in the absence of matched tumor DNA. In addition, analysis of cfDNA for somatic mutations may be affected by the presence of mutations that do not arise from the tumor. These include germline mutations, mutations resulting from clonal events in non-cancerous tissue, and sequencing artifacts (11). The most common clonal mutations originate in the hematopoietic system and may be misclassified as tumor mutations (25).

CH occurs naturally as a result of the accumulation of somatic mutations and the clonal proliferation of hematopoietic stem cells with normal aging. The identification of these CH-mutations has been described as a source of biological noise in blood liquid biopsy (6).

The idea that CH mutations may be detected in plasma cfDNA was first proposed in two lung cancer studies (9, 26). Up to 15% of TP53 mutations found in lung cancer patients' plasma cfDNA were also found in white blood cells, indicating a CH origin. These early findings were confirmed in a prospective study that used a large gene panel and performed deep sequencing of cfDNA and matched white blood cells from patients with metastatic cancers (27). The study by Razavi et al. (27) showed that the majority of cfDNA mutations seen in cancer patients originate in white blood cell clones, and not in the tumor. Interestingly, the authors noted that the majority of mutations detected by the cfDNA analysis were coming from white blood cell clonal expansions in both control and cancer patients. As a result, since mutations in hematopoietic clones and solid tumors may be similar, sequencing cfDNA alone may not be enough to differentiate malignancy from CH. Even in individuals with metastatic disease, the number of mutations generated from CH was on average greater than the number of variants acquired from tumors (27).

Liquid biopsy was recently approved to detect actionable mutations in certain genes that may aid in treatment selection in the absence of tumor tissue. CH mutations should be carefully examined in these assays since recent studies have revealed that a significant amount of CH mutations can be oncogenic and actionable (9, 27, 28). According to a study by Razavi et al., up to 10% of CH mutations detected in plasma were classified as oncogenic, and 13% of these mutations were related with an approved targeted therapy or a clinical trial treatment. Incorrect classification of cfDNA mutations would result in ineffective therapy. Furthermore, CH interference can cause false-positive cfDNA biomarker assessments that may result in inappropriate treatment decisions. For example, in a cohort of patients with advanced prostate cancer (29), 10% of the patients had CH interference in plasma cfDNA in DNA repair genes that are used for eligibility of PARPi therapy, including *ATM, BRCA2*, and *CHEK2*.

The minimally invasive nature of liquid biopsy and the ability for serial sampling makes liquid biopsy an ideal tool for detecting MRD and monitoring therapy response in patients with GI malignancies (16, 30, 31). However, the majority of previous research did not remove from their analyses CH mutations which can be a source of biological noise.

## Clinical Impact of Clonal Hematopoiesis Mutations on the Interpretation of Liquid Biopsy Results in Patients With Gastrointestinal Cancers

Recently, several studies have been performed to evaluate the identification of CH mutations in plasma and their impact on the interpretation of blood liquid biopsy results in patients with GI cancers (Table 1). A list of genes included in the panels from the studies mentioned above is available as a supplementary material (Supplementary Table 1).

**Table 1:** Summary of published studies on the prevalence of CH mutations from plasma cfDNA analysis in patients with GI cancers.

**Study**	**Study population**	**Cancer type**	**Sequencing method**	**Gene panel**	**Prevalence of CH detection from plasma cfDNA analysis**
Chan et al. (32)	38	Colorectal cancer	Targeted NGS	52 genes	**17%**
Leal et al. (28)	50	Gastric cancer	Targeted NGS	58 genes	**52%**
Huang et al. (33)	236	Metastatic colorectal cancer	Targeted NGS and ddPCR	4 genes	**1.27%**
Ococks et al. (35)	97	Esophageal cancer	Targeted NGS	77 genes	**23%**

Chan et al. (32) evaluated the impact of CH-related mutations on the clinical interpretation of liquid biopsy in a cohort of colorectal cancer patients. Seventeen percentage of the mutations detected in the pre-operative cfDNA were CH-related. In this study, the patients were monitored post-operatively and the CH mutations discovered on a recurrent basis following surgery or adjuvant chemotherapy completion. Without paired-end sequencing of cfDNA and white blood cells DNA, the persistent detection of CH -related mutations in plasma samples could be misinterpreted as disease progression or treatment inefficacy.

Another recent study aimed to determine how KRAS, NRAS and BRAF mutations in hematopoietic cells may alter the results of liquid biopsies in patients with metastatic colorectal cancer (33). The authors also performed a comparison between patients with and without prior chemotherapy to assess the contribution of chemotherapy to CH. The prevalence of CH was 1.27%, and cfDNA mutations originating from CH persisted during treatment, unlike tumor-derived mutations, which decreased over time. Although the prevalence of CH was limited in this colorectal cancer patients population, the study provided further evidence for the importance of gDNA genotyping in peripheral blood cells (PBC) when performing cfDNA analysis in plasma. Another important finding of this study is that the CH -derived mutations identified were clustered in a particular subgroup of patients that had 5% allele frequency mutations. This subgroup received prior chemotherapy, suggesting that pre-selection of patients may improve the results of PBC gDNA genotyping.

The impact of CH on the interpretation of liquid biopsy results was also evaluated in patients with gastric cancer. In a study by Leal et al. (28), a matched cfDNA and white blood cell sequencing method was used to characterize ctDNA changes after preoperative chemotherapy and/or surgery in patients with resectable gastric cancer. At baseline, mutations were detected in the cfDNA of 40 patients (80%) and in the leukocytes of 31 patients (62%). After subtracting the leucocytes-derived alterations from the cfDNA data, 54% of patients were found to have alterations that were likely tumor-specific. According to these results, cfDNA testing alone would not have been able to adequately identify patients benefiting from perioperative treatment in terms of progression-free and overall survival.

There have been relatively few data on the use of ctDNA in resectable esophageal adenocarcinoma. One study (34) examined a cohort of 22 resected esophageal cancer patients using a commercially available ctDNA panel and discovered that four of seven patients with positive ctDNA had recurrence after surgery. CH mutations were not excluded from the analysis, and the authors reported that three elderly patients who were ctDNA positive and had longer disease-free survival may have had false positive results due to CH.

A recent study by Ococks et al. (35) evaluated the prognostic value of ctDNA in a prospective cohort of patients with resectable esophageal adenocarcinoma. The authors demonstrated that detection of ctDNA following curative resection is associated with an increased risk of cancer recurrence and a decreased survival rate. Moreover, patients without ctDNA detected postoperatively had a 2-fold increase in cancer-specific survival compared to those who were ctDNA-positive. According to this study, eliminating false-positive readings by excluding CH-derived variants improves the specificity and predictive value of ctDNA in this setting.

**Figure 1:**
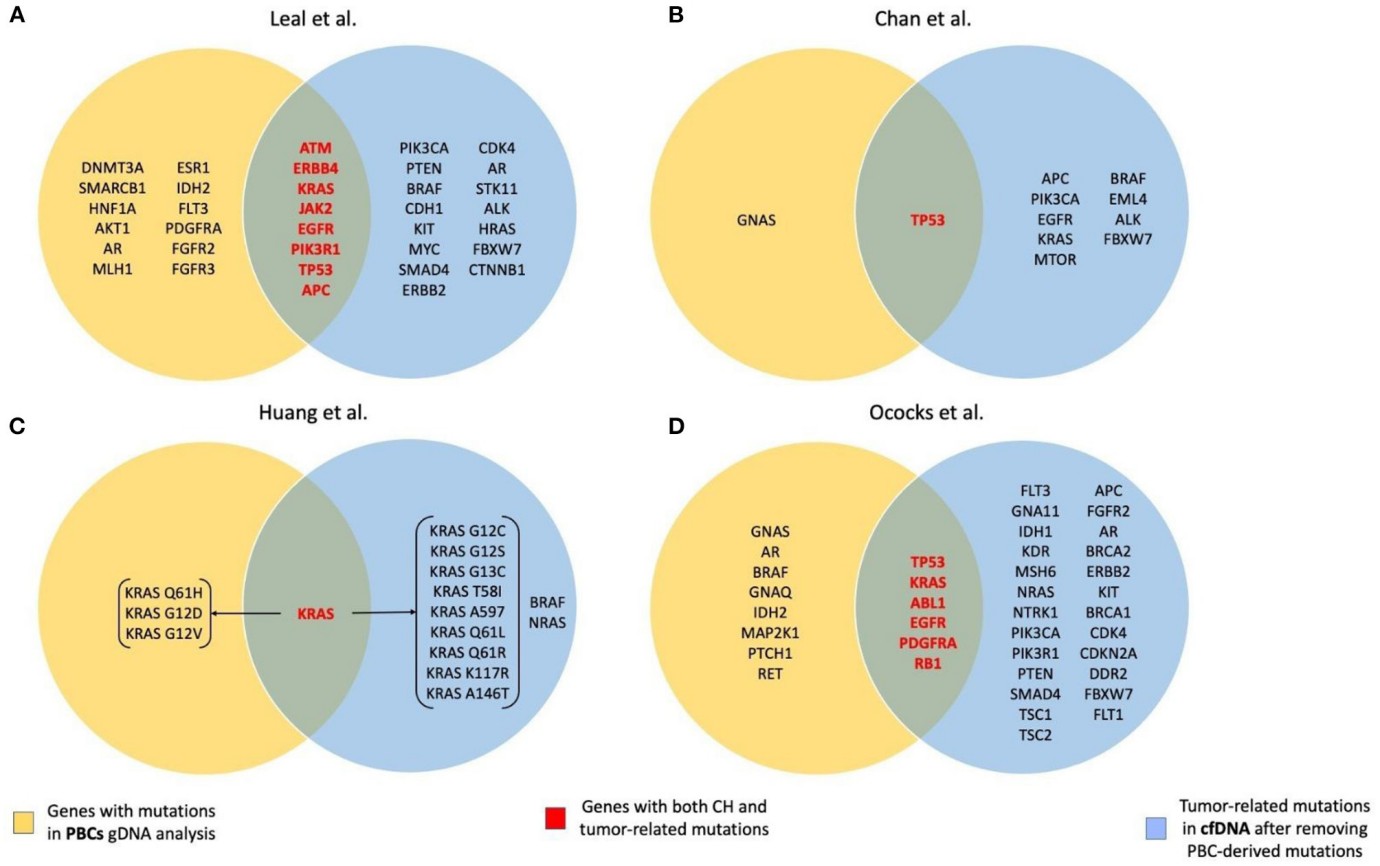
Summary of the genes with CH and tumor-related mutations found in the studies included in the review. **(A)** Leal et al. (28); **(B)** Chan et al. (32); **(C)** Huang et al. (33); **(D)** Ococks et al. (35).

A summary of genes with CH and tumor-related mutations found in the studies included in the review is presented in Figure 1.

## Discussion

cfDNA liquid biopsies may be a useful tool for the evaluation of predictive and prognostic biomarkers, therapy resistance, and detection of MRD after surgery in patients with GI cancers. However, special care should be taken in order to determine the origin of genetic mutations and to distinguish tumor-derived ctDNA from other sources of genetic alterations present in the blood, such as CH mutations.

Recent data indicated that CH is more frequent in patients with solid cancer, with ~30% of them having CH mutations in their blood (36). Cancer patients may have higher rates of CH than their normal same age group counter-parts due to increased environmental carcinogenic exposures and genotoxic therapies (37). With the recent increase of liquid biopsies in GI malignancies the chance of also finding CH-related mutations did also increase. The detection of CH is of particular importance in the clinical application of liquid biopsy for the detection of MRD and in cancer screening, where false positives due to CH can lead to erroneous and harmful conclusions.

On the other hand, the clinical risk associated with CH is not yet clear. There is evidence of an increased risk of myeloid neoplasms (36), cardiovascular disease, ischemic stroke and heart failure (7, 37, 38). These pathological associations underscore the potential clinical significance of CH detected from liquid biopsy. Bolton et al. (39) highlighted the relevance of CH as a predictor and precursor of therapy-related myeloid neoplasms (tMN) in patients with cancer. The authors demonstrated that radiation therapy and cytotoxic therapies were significantly associated with CH, with regimens containing platinum and topoisomerase II inhibitors most strongly correlating with CH in specific DNA damage response pathway genes including TP53, PPM1D and CHEK2. Serial sampling before and after therapy revealed that therapy causes gene-specific clonal expansion, in which clones with DDR gene mutations outcompete other clones in the context of cancer therapy. Mutations in these genes are linked to chemo-resistance and are highly enriched in tMN.

Therefore, CH should not be considered only as a source of biological noise in cfDNA analysis, as these mutations could also provide valuable clinical information. Long-term consequences of CH are of a lesser concern for patients with incurable metastatic tumors or with a poor prognosis. In these patients population, CH may be the herald of more severe chemotherapy side effects, such as marrow suppression from cytotoxic therapy. On the other hand, for patients undergoing adjuvant therapy with curative intent, there may be scenarios in which the risk of exacerbating CH outweighs the risk of cancer recurrence (37).

The hematopoietic system plays an important role in regulating inflammation and immunity. CH has been related to a number of negative health outcomes, with inflammation emerging as a significant mediator (40). Dharan et al. (41) suggested that chronic HIV infection, might be an etiology for CH, whereas Bolton et al. (42) established the relationship of CH with a wide spectrum of infections, including infection with SARS-CoV. Inflammation and clonal growth are reinforcing one other in a self-perpetuating circle. Thus, low-grade inflammation promotes clonal proliferation of specific mutant hematopoietic stem cells, which, as the clone size increases, promotes inflammation further, creating a vicious loop that eventually affects organ function (43). The current method for identifying CH is through performing paired sequencing of plasma cfDNA and white blood cell DNA. However, the additional costs associated with paired sequencing could act as a barrier to the routine use of liquid biopsy in clinical practice. A better understanding of the molecular characteristics of CH combined with machine learning may eliminate the need for white blood cell-paired sequencing (44).

**Figure 2:**
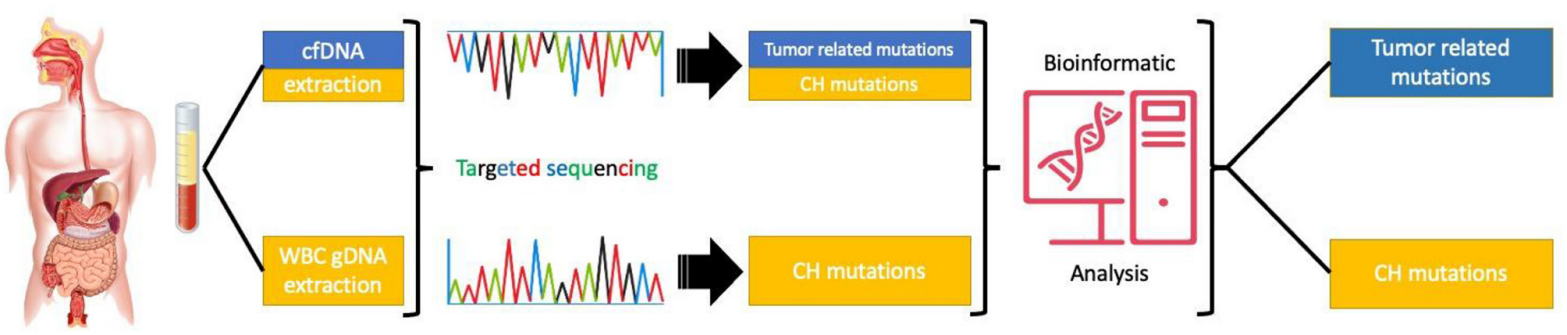
Targeted sequencing of paired cfDNA and white blood cells to correctly asses the origin of the mutations.

In conclusion, CH mutations may have a significant contribution to the amount and nature of mutations identified in the peripheral blood of patients with GI malignancies. In order to avoid mismanagement of cancer patients, protocols distinguishing tumor-derived cfDNA mutations from CH related genetic alterations should be introduced in routine clinical practice (Figure 2).

## Author Contributions

VC, AC, and AT: conception and design of the work. VC, IC, and IP: literature review and data collection. VC and IC: drafting the article. DP, SD, AT, and AC: critical revision of the article. All authors have read and agreed to the current version of the manuscript.

## Funding

This work was supported by grant of Ministery of Research and Innovation, CCCDI UEFISCDI, under project number PN-III-P4-ID-PCCF2016-0158, contract number PCCF17/2018 within PNCDI III.

## Conflict of Interest

The authors declare that the research was conducted in the absence of any commercial or financial relationships that could be construed as a potential conflict of interest.

## Publisher's Note

All claims expressed in this article are solely those of the authors and do not necessarily represent those of their affiliated organizations, or those of the publisher, the editors and the reviewers. Any product that may be evaluated in this article, or claim that may be made by its manufacturer, is not guaranteed or endorsed by the publisher.
