# Supplementary material for: Clonal Hematopoiesis and Liquid Biopsy in Gastrointestinal Cancers
Source: Front Med (Lausanne). 2022 Jan 21;8:772166. doi: 10.3389/fmed.2021.772166 (PMC8814311; doi:10.3389/fmed.2021.772166)
Supplement: Supplementary file 1 [file Table_1.DOCX]

| Leal et al. ^28^ | Chan et al. ^32^ | Huang et al. ^33^ | Ococks et al. ^35^ |
| --- | --- | --- | --- |
| ABL1, AKT1, ALK, APC, AR, ATM, BRAF, CDH1, CDK4, CDK6, CDKN2A, CSF1R, CTNNB1, DNMT3A, EGFR, ERBB2, ERBB4, ESR1, EZH2, FBXW7, FGFR1, FGFR2, FGFR3, FLT3, GNA11, GNAQ, GNAS, HNF1A, HRAS, IDH1, IDH2, JAK2, JAK3, KDR, KIT, KRAS, MAP2K1, MET, MLH1, MPL, MYC, NPM1, NRAS, PDGFRA, PIK3CA, PIK3R1, PTEN, PTPN11, RB1, RET, SMAD4, SMARCB1, SMO, SRC, STK11, TERT, TP53, VHL | AKT1, ALK, APC, AR, ARAF, BRAF, CCND1, CCND2, CCND3, CDK4, CDK6, CHEK2,  CTNNB1, DDR2, EGFR, ERBB2, ERBB3, ERG, ESR1, ETV1, FBXW7, FGFR1, FGFR2, FGFR3, FGFR4, FLT3, GNA11, GNAQ, GNAS, HRAS, IDH1, IDH2, KIT, KRAS, MAP2K1, MAP2K2, MET, MTOR, MYC, NRAS, NTRK1, NTRK3, PDGFRA, PIK3CA, PTEN, RAF1, RET, ROS1, SF3B1, SMAD4, SMO, TP53 | BRAF, KRAS, NRAS, PIK3CA | ABL1, AKT1, AKT2, ALK, APC, AR, ARAF, BRAF, BRCA1, BRCA2, CCND1, CCND2, CCND3, CD274, CDK4, CDK6, CDKN2A, CSF1R, CTNNB1, DDR2, DPYD, EGFR, ERBB2, ESR1, EZH2, FBXW7, FGFR1, FGFR2, FGFR3, FLT1, FLT3, FLT4, GATA3, GNA11, GNAQ, GNAS, IDH1, IDH2, JAK2, JAK3, KDR, KEAP1, KIT, KRAS, MAP2K1, MAP2K2, MET, MLH1, MSH2, MSH6, MTOR, NF2, NFE2L2, NRAS, NTRK1, PDCD1LG2, PDGFRA, PDGFRB, PIK3CA, PIK3R1, PMS2, PTCH1, PTEN, RAF1, RB1, RET, RNF43, ROS1, SMAD4, SMO, STK11, TERT, TP53, TSC1, TSC2, UGT1A1, VHL |

**Supplementary Table 1** – List of genes included in the NGS panels for each study
